# Oxygen “therapy” for infection in liver transplant surgery: less is more, enough is enough

**DOI:** 10.1186/s12916-023-02767-0

**Published:** 2023-02-13

**Authors:** Cheng-Maw Ho, Hsin-Yun Sun

**Affiliations:** 1 Department of Surgery, National Taiwan University Hospital and College of Medicine, 7 Chung-Shan South Road, Taipei, 100 Taiwan; 2 Department of Internal Medicine, National Taiwan University Hospital and College of Medicine, Taipei, Taiwan

**Keywords:** Liver transplantation, Infection, Oxygen, Surgery

## Background

The establishment of infection relies on the close interaction of the host, microbes, and the in-between conductive environment. In the setting of liver transplantation, the early postoperative period carries a particularly high risk of infection, because the host had just gone through extensive surgical stress and received high degrees of immunosuppression. During the early post-transplantation period, healthcare-acquired bacterial and fungal infections are the most common types of infection encountered in liver transplant recipients [1]. Because the World Health Organization guidelines for reducing surgical site infection have recommended the perioperative administration of high-dose oxygen [2], the issue of high-dose oxygen therapy for infection in liver transplantation raises interests.

## Main text

### Oxygen “therapy” for infection in liver transplantation

Suzuki nicely summarized the pros and cons of oxygen administration in perioperative periods [3]. Most postoperative surgical patients routinely receive supplemental oxygen therapy to prevent the potential development of hypoxemia due to incomplete lung re-expansion, reduced chest wall, and diaphragmatic activity caused by surgical site pain, consequences of hemodynamic impairment, and residual effects of anesthetic drugs (most notably residual neuromuscular blockade), which may result in atelectasis, ventilation–perfusion mismatch, alveolar hypoventilation, and impaired upper airway patency [3]. However, supplemental oxygen and hyperoxemia in perioperative periods can have harmful effects on the respiratory and cardiovascular systems and on associated adverse clinical outcomes by inducing absorption atelectasis [4], impeding minute ventilation in spontaneously breathing subjects, worsening ventilation–perfusion matching, shifting the carbon dioxide dissociation curve to the right (Haldane effect) [5], and reducing cardiac output and increasing systemic vascular resistance [6]. Although the updated evidence showed that high-dose oxygen had weaker benefits and the new guidelines downgraded the strength of the recommendation from strong to conditional, the general recommendation to ventilate intubated surgical patients with an FiO2 80% was retained [2, 3].

In Figiel et al.’s study [7], they aimed to reduce postoperative infection by giving a supplemental high concentration of oxygen for liver transplant recipients in the early postoperative period. A total of 193 patients was randomized into two groups with FiO2 80% vs. 28% in the early postoperative period for 6 h. For patients under mechanical ventilation continued in the postoperative period, it was straightforward. For extubated patients assigned to the 80% group, oxygen had to be delivered by 14 L/min of oxygen and 2 L/min of air through a non-rebreathing face mask with a reservoir [7]. The occurrence of infection in the 30-day postoperative period was the primary outcome measure, and the 90-day severe morbidity rate was one among the secondary outcome measures [7]. The hypothesis was that provision of 80% FiO2 reduces the risk of postoperative infections from 40 to 24%. Although there was early study termination for the outbreak of COVID-19, surprisingly, the results were in the opposite direction.

Postoperative infections in the 30-day post-transplant period, as the primary outcome measure, were higher in the 80% group (34% vs. 23.2%, no statistical significance) as compared to the 28% group [7]. Moreover, the 80% group suffered more frequently severe complications (43.6% vs. 28.3%) in the 90 days after transplantation, stayed longer in the intensive care unit, and had higher bilirubin concentration over the first 5 post-transplant days [7]. Conversely, a more detailed comparison revealed that the increased severe morbidity rate in the 80% group was largely due to a higher number of complications potentially caused by technical reasons, such as hemorrhage, hepatic artery thrombosis, and biliary leaks [7]. Therefore, despite that severe complications were significantly more frequent in patients assigned to 80% FiO2, Figiel et al.’s study does not prove the direct causative effect of high FiO2 on severe morbidity [7].

The study design of the trial is worth further discussion. On the paper they based, Belda et al. designed the randomization in the perioperative period (including the operative and postoperative periods), not just the postoperative period [8]. We all know that during surgery, oxygen demand may be increased and hemodynamic changes may compromise tissue oxygenation in this regard, posing a risk of infection if low oxygen supply. Only 6 h after the postoperative period is quite a short time as a therapeutic intervention, and the potential frequent changes of respiratory modalities (early or late extubation and using a non-rebreathing mask to keep 80% oxygen or definite 80% by machine respirator), and bias by swift shifting of oxygen supply from the operative period to the postoperative period (which was not addressed in this paper), may actually contribute to the contrary results.

## Conclusions

Routine high-dose oxygen supplementation is ineffective in the prevention of infections and should be avoided during postoperative settings in liver transplantation. Management of postoperative hypoxemia counts more than routine postoperative hyperoxemic oxygen “therapy.” Less is more and enough is enough.

## Data Availability

Not applicable.
